# Using Prevention Research to Reduce Racial Disparities in Health Through Innovative Funding Strategies: The Case of Doula Care

**DOI:** 10.1007/s11121-023-01497-2

**Published:** 2023-02-09

**Authors:** Judy A. Temple, Nishank Varshney

**Affiliations:** Humphrey School of Public Affairs and Human Capital Research Collaborative, University of Minnesota - Twin Cities, 301-19th Avenue South, MN 55455 Minneapolis, USA

**Keywords:** Health disparities, Doulas, Economic evaluation, Prevention, Pay for success, Social impact bonds, Data-driven philanthropy

## Abstract

Racial disparities in maternal birth outcomes are substantial even when comparing women with similar levels of education. While racial differences in maternal death at birth or shortly afterward have attracted significant attention from researchers, non-fatal but potentially life-threatening pregnancy complications are 30–40 times more common than maternal deaths. Black women have the worst maternal health outcomes. Only recently have health researchers started to view structural racism rather than race as the critical factor underlying these persistent inequities. We discuss the economic framework that prevention scientists can use to convince policymakers to make sustainable investments in maternal health by expanding funding for doula care. While a few states allow Medicaid to fund doula services, most women at risk of poor maternal health outcomes arising from structural racism lack access to culturally sensitive caregivers during the pre-and post-partum periods as well as during birth. We provide a guide to how research in health services can be more readily translated to policy recommendations by describing two innovative ways that cost–benefit analysis can help direct private and public funding to support doula care for Black women and others at risk of poor birth outcomes.

## Introduction

Racial health disparities are observed across various health outcomes. One notable area of disparity is maternal birth outcomes, where the differences across races are huge even when comparing women with similar levels of education. Black women are over three times more likely than White women to experience pregnancy-related death. While racial differences in maternal death at birth or shortly afterward have attracted significant attention from researchers in a variety of disciplines (Bridges, 2020; Chinn et al., 2020; Scholars, 2020), the number of non-fatal but potentially life-threatening complications of pregnancy are 30–40 times more common than maternal deaths. These severe maternal morbidity events also exhibit racial disparities putting Black women at higher risk than White women and others. Moreover, racial differences also exist in infant mortality and unintended birth-related outcomes such as preterm birth and cesarean deliveries (Mathews et al., 2015; Tangel et al., 2019).

Racial disparities in health outcomes in the USA have existed for hundreds of years (Thomas & Casper, 2019), but health researchers have only recently started to view structural racism rather than race as the critical factor underlying these persistent inequities (Crear-Perry et al., 2021; Scott & Davis, 2021). Hardeman and Karbeah (2020) find that there is insufficient attention on the role of structural racism in access to quality education, residential location, and underinvestment in health services and other community resources affecting Black populations. We discuss the stark differences in health outcomes for Black mothers before and during childbirth and the post-partum period and highlight innovative funding mechanisms involving public/private partnerships that use economic evaluation tools to better expand programs that help address these inequities.

The targeted expansion of spending on preventive interventions can potentially reduce racial disparities in health outcomes. We focus on the case of doula support for Black women in low-income communities as an example of how increased spending may help offset societal risks to Black women’s health as well as those risks that Scott and Davis (2021) refer to as obstetric racism. Doulas are trained professionals who offer informational guidance and physical and emotional support to pregnant mothers before and after childbirth and can act as an intermediary between the mother and other medical personnel.

We illustrate how two innovative financing methods, Pay for Success and data-driven philanthropy, can direct additional resources to reduce these racial disparities. We employ an economic framework recommended in a report by the National Academies of Sciences, Engineering, and Medicine (2016) for valuing the benefits and costs of preventive programs. The first approach is based on what prevention scientists Crowley and Jones (2017) describe as a framework for “valuing investments for a nurturing society,” in which increased spending on preventive interventions is motivated by highlighting the cost savings to taxpayers. We also introduce a second funding strategy that focuses entirely on the benefits to participating individuals without considering benefits to taxpayers or other third parties. Both applications of economic evaluation can enable policymakers to make sustainable investments in maternal health by leveraging private–public partnerships to expand doula care to serve Black communities.

## Racial Differences in Maternal Mortality, Maternal Morbidity, and Infant Health

Racial disparities in maternal mortality in the USA are substantial. While studies in other countries suggest that mortality has fallen over time (Alkema et al., 2016), pregnancy-related mortality in the USA is rising (MacDorman et al., 2017). The most recent analysis of national data from the Centers for Disease Control (CDC) suggests an overall pregnancy-related mortality rate (during or within 1 year of pregnancy) of 16.7 per 100,000 live births from 2007 to 2016. Black women had a mortality rate of 40.8 compared to White, Hispanic, and Indigenous women (12.7, 11.5, 29.7, respectively) (Petersen et al., 2019). For the remainder of this paper, we focus on racial disparities involving Black women. In their analyses using US states that potentially may have better quality data and a more restrictive definition of death within 42 days of birth, MacDorman et al. (2017) report higher mortality rates, with Black women having 56.3 deaths per 100,000 live births in 2013–2014. While there is uncertainty about the precise rates, there is no dispute that mortality rates for Black women are stunningly high.

While maternal mortality rates are higher for those with less than a college education, unmarried status, and older than 40 (Petersen et al., 2019), studies that control these risk factors still find sizeable racial gaps. In Table 1, we highlight a few findings from CDC analyses of national data for 2007–2016, where pregnancy-related mortality rates (PRMR) by race are shown disaggregated by levels of educational attainment as well as state-wide maternal mortality levels.

**Table 1:** Pregnancy-related mortality rates per 100,000 live births by race and ethnicity, education, and state-wide mortality levels, 2007–2016

Pregnancy-related mortality rate (PRMR)	Total	Black	Indigenous^a^	Hispanic	White
Total	16.7	40.8	29.7	11.5	12.7
By education completed					
Less than high school	21.6	45.6	50.8	12.6	25.0
High school	27.4	59.1	43.7	11.2	25.2
Some college	16.4	41.0	32.0	9.4	11.7
College graduate or higher	10.9	40.2	-^b^	9.3	7.8
State-wide PRMR rates^c^					
Lower third of states^d^	10.7	26.0	28.9	9.7	8.7
Upper third of states	21.9	45.9	28.8	13.2	16.6

Data from Petersen et al. (2019)

^a^Indigenous include American Indian and Alaska natives

^b^Fewer than 10 deaths in that category

^c^State-wide rates include all races and ethnicities

^d^The third of states with the lowest overall pregnancy-related mortality rates in the USA

Mortality rates are highest for mothers who did not attend college, but notably, for Black women, even those who attended college have significantly higher PRMR than White or Hispanic women. For Black women who graduated from college and may have advanced degrees, the mortality gap remains substantial, having five times the risk of death compared to White women. There is also a significant variation in death rates across states. The PRMR for Black women increases significantly in states where the overall mortality rate is high. But even among the states with the lowest overall PRMR, Black women have over 2.5 times the risk of death than average. These findings suggest that local healthcare and environmental contexts are important.

While racial disparities in maternal deaths are striking, maternal morbidity is much more common and exhibits similarly high rates for Black women. Severe maternal morbidity (SMM) occurs when a mother experiences a life-threatening condition or requires a lifesaving procedure during birth. In 2017, severe morbidity incidents were reported in over 25,000 hospital births (Leonard et al., 2019), including hysterectomy, eclampsia, clotting/bleeding disorders, cerebrovascular and cardiovascular complications, and kidney and respiratory failure.

Even though severe maternal morbidity rates are around 30–40 times higher than mortality rates, there appear to be fewer comprehensive assessments by race and ethnicity. Studies using national in-patient surveys report higher morbidity rates for Black women for some specific pregnancy complications. Miller et al. (2020) look specifically at cerebrovascular complications or strokes and find significantly higher rates of strokes for pregnant Black women. Similarly, Gad et al. (2021) report higher rates of cardiovascular disease events for Black relative to White women during pregnancy, even controlling for hypertensive status. Hospitalizations from SMMs can occur at various times during pregnancy. Liese et al. (2019) examined national data focusing on hospitalized pregnant women and reported significantly higher odds for Black women to be hospitalized for SMM during the antepartum and post-partum periods.

Significant racial disparities exist in prematurity, where the preterm birth rate for Black women is 1.6 times higher than for White women (Mathews et al., 2015). This is the leading cause of death in Black infants, who are more than twice as likely to die in the first year of life than White infants (Mathews et al., 2015). These disparities exist even in states with high average income and education levels. In Minnesota, for example, Black infant mortality rates of 9.8 per 1000 are more than twice the rate of 4.4 per 1000 for White births (Minnesota Department of Health, 2015). The same report found that infants born to college-educated Black women had a higher risk of death than White women who were not high school graduates.

### Poor Maternal Health Outcomes and Structural racism

Living in a low-income household, having fewer years of formal education, lacking or having discontinuous coverage in health insurance, and living in a high-poverty neighborhood with chronic stress and a lack of access to healthy foods can be considered risk factors for pregnancy complications (Bridges, 2020; Petersen et al., 2019). The fact that these socio-economic conditions, as well as pre-pregnancy health conditions such as hypertension or obesity, vary by race has, for decades, led researchers and the popular press to view race as a risk factor for a healthy pregnancy.

While race has long been discussed as a risk factor for poor maternal health outcomes, it is now being recognized that structural racism rather than race is a significant causal factor behind these inequities. Hardeman and Karbeah (2020), Bridges (2020), and others are calling attention to the impact of structural racism on access to good schools, residential locations, and the under-investments in health and other community resources affecting Black populations. In June 2020, the Society for Prevention Research (SPR) policy statement condemning anti-Black racism recognized “institutional, systemic, and individual racism as a fundamental cause of disparities across all of society, directly affecting health, housing, education, employment, and equitable treatment of Black Americans” (SPR, 2020). The American Public Health Association (APHA) also issued a statement describing structural racism as a public health crisis and explaining that it “differentially distributes the goods, services, opportunities, and protections of society by race, including safe and affordable housing, quality education, adequate income and wealth-building, employment, accessible quality health care, and healthy neighborhoods” (APHA, 2020).

Viewing racial disparities in maternal health outcomes through the lens of structural racism significantly alters the scope and scale of potential policy remedies. For example, viewing hypertension and obesity as resulting from chronic stress and inadequacies in the built environment that limits physical activity and access to healthy foods or considering in-patient outcomes as specific to the hospital (Janevic et al., 2020) places policy attention on how and why the built environment or health care system has these deficiencies that fail to serve Black families. A recent study by Greenwood et al. (2020) showed significantly lower rates of Black infant deaths when care was provided by a Black physician vs. a White physician within the same hospital. The authors suggest that implicit bias plays a role.

Viewing race-related risks as the result of inequities in the distribution of society’s resources, including access and quality of health care, can provide stronger motivation for investments in prevention. Phibbs et al. (2022) explicitly refer to recent studies of hospital quality improvement efforts as a rationale for concluding that many SMM events are preventable, while others, such as Janevic et al. (2020) and Petersen et al. (2019), refer to evidence from state mortality review committees and other sources to suggest that at least half of maternal deaths and at least a third of SMM events are preventable (e.g., Zuckerwise & Lipkind, 2017).

## Doula Care as Means of Improving Maternal and Child Health Outcomes

Reductions in racial disparities in maternal and infant health outcomes could potentially be achieved by providing culturally sensitive labor support to expectant mothers through doula care. DONA International, the largest international organization for doulas, defines a doula as “a trained professional who provides continuous physical, emotional, and informational support to a mother before, during, and shortly after childbirth to help her achieve the healthiest, most satisfying experience possible” (DONA International, n.d.). Typically, a doula meets with an expectant mother two to four times in the prenatal period, stays with the mother during labor and at least 1 h after the birth, and meets with the new mother one to two times in the postnatal period (Gruber et al., 2013). Doulas may or may not have formal training, although DONA International requires 16 h of training for certification. Gebel and Hodin (2020) estimate that there may be well over 80 organizations across the USA that train or certify doulas.

Gebel and Hodin (2020) describe several types of doulas. Birth doulas support clients during pregnancy, birth, and into the early post-partum period. Post-partum doulas offer care in the weeks or months after delivery. In contrast, community-based doulas serve clients in under-resourced communities and may take a broader interest in health issues to help reduce workloads for nurses and other clinical care providers.

Doulas have been found to be effective in improving maternal post-partum health outcomes. Many randomized control trials (RCTs) have investigated outcomes associated with continuous, one-to-one support during labor as compared to regular care. Zhang et al. (1996)’s meta-analysis was based on four randomized studies across multiple countries that focused on low-income, inner-city women. This meta-analysis concluded that women with continuous support have lower c-section rates, shorter labors, and report higher satisfaction with their hospital care. More recently, Bohren et al. (2019) conducted a detailed summary of 26 RCTs in high- and middle-income countries and concluded that continuous support is positively associated with reductions in c-section rates, fewer babies with low Apgar scores, and fewer adverse reports about patient care. However, there may be concerns about the quality of the evidence as the experiments are never blind, sample sizes may be too small to identify impacts on some outcomes, and the studies vary in terms of the care and support that the women in the comparison group receive. In two RCT studies of doula care in the USA, researchers found that differences in c-sections or preterm births favored doula-supported women, but these differences were not statistically significant (Campbell et al., 2006; Hans et al., 2018). Campbell et al. (2006) (*N* = 598; 36% Black, 56% White) emphasized that their study location (a university-affiliated hospital offering high levels of medical intervention) may have limited their ability to find the effects of support during labor. In the RCT of home-visiting doulas studied by Hans et al. (2018) (*N* = 312; 45% Black, 8% White), multiple contacts by case managers for both treatments and controls could help explain the smaller effects of doulas.

Additional evidence on doula care effectiveness comes from non-experimental studies comparing women who choose a doula versus those who do not. Everson et al. (2018) compared the outcomes from 1892 doula-supported adolescent births in the USA (20.7% Black, 45.9% White) with national statistics and found lower rates of c-section and preterm births in the doula-assisted group. In a non-experimental study of 226 women (78% Black, 7% White), Gruber et al. (2013) found that doula care is associated with a reduction in low birth weight, higher rates of breastfeeding initiation, lower rates of births with complications, and reductions in c-section births. Kozhimannil et al. (2016) use regression analysis, include pregnancy-induced hypertension and diabetes as controls, and find that doula-supported women had 22% lower odds of preterm birth and significantly lower odds of cesarean delivery among full-term births (*n* = 1935, 47.2% Black, 10.3% White).

Research has also found doula care to impact maternal mental health. Randomized controlled studies conducted in South Africa found that women with doula support reported lower post-partum depression and anxiety ratings at 6 weeks (Wolman et al., 1993) and 12 weeks after delivery (Trotter et al., 1992). In the USA, researchers reported that mothers randomly assigned access to post-partum doulas versus peer support were more satisfied with receiving assistance from doulas (Gjerdingen et al., 2013). Using propensity score matching, Falconi et al. (2022) found that women whom doulas supported during labor and birth reported lower rates of post-partum depression or anxiety. In a qualitative observational study, McComish et al. (2013) concluded that women build a bond with the doulas before birth, allowing them to share their feelings of depression and follow advice for seeking mental health treatment.

Overall, results from numerous studies provide evidence that doula care can impact important maternal health outcomes and reduce rates of preterm births. To date, studies have not been large enough to detect differences in maternal death and severe morbidity events arising from doula care. Some of the RCTs, as mentioned, have limitations favoring the finding of smaller impacts. Overall, a summary of the evidence suggests that, through providing support and health care information, doulas can reduce the likelihood of poor outcomes that contribute to racial disparities in health.

### Public Funding for Doula Care

The supply of doulas is a concern. It has been estimated that doulas serve only 6% of all pregnancies. Efforts to increase the supply of culturally sensitive Black doulas could benefit Black women of all income levels. How much does a doula cost? One recent report estimated the cost to be $800 to $2500 per pregnancy (Weiss, 2021). The cost associated with training, certification, and being registered with the state limits diversity in the doula workforce (Kozhimannil et al., 2015; Lantz et al., 2005; Van Eijk et al., 2022a, 2022b), which is a concern for women desiring culturally sensitive care and those who have experienced discrimination from health care providers. Doula training programs can cost several thousand dollars, and the required time spent shadowing and engaging in additional activities to become certified is often unpaid (Van Eijk et al., 2022a, 2022b). While some private and public insurance plans pay for doula care, some women pay without billing insurance (DeClercq et al., 2014), and some doulas work as volunteers.

Given that over 40% of all births in the USA are paid for by Medicaid (a program requiring both state and federal taxpayer contributions to fund care for low-income patients), policymakers in several states have recognized that providing better care to pregnant women may benefit taxpayers as well. The Minnesota state government allows for the billing of care by a certified doula as long as that person is supervised by a physician, nurse practitioner, or certified nurse wife. As of 2022, the payment rate is $47 per pre-or post-partum visit with assistance at the birth, billable for $488. Given a maximum of six allowable non-birth visits, the total amount that doulas can bill for is $770. In Oregon, the state allows reimbursement for two prenatal visits, two post-partum visits, and labor and delivery for a total rate of $350 (Kozhimannil & Hardeman, 2016). This rate of $350 has remained constant, but in 2022, a state agency announced plans to seek an increase to $1500 per pregnancy (Oregon Health Authority, 2022). Platt and Kaye (2020) describe strategies used in Nebraska and Indiana that use federal block grant funds to support a program offering doula care. New Jersey recently began Medicaid funding for doula care and has higher reimbursement rates. It incentivizes the scheduling of more doula visits—as of 2022, doulas can bill $66 per visit for a total of 12 pre-or post-partum visits in addition to attendance at the birth billed at $235, at a total of $1066 per birth (State of New Jersey, 2021). Chen (2018) provides guidance for advocates and policymakers on expanding the number of states that use Medicaid for this purpose.^1^

Even when reimbursement is possible from private or public insurance, low reimbursement rates limit possibilities for expanding and sustaining the workforce (Kozhimannil & Hardeman, 2016), especially when combined with the need for costly training, ongoing certification, and registration costs. Gomez et al. (2021) interviewed doulas at a community health center that were either paid a flat fee per client or paid hourly with benefits and found that the former resulted in especially inequitable working conditions. Expanding and diversifying the doula workforce will require better compensation.

### Economic Evaluation of Doula Care Programs

An economic evaluation of doula care first requires evidence of effectiveness and then involves calculating the costs of generating the outcomes of interest. As described above, doula care has been found to be effective in improving maternal and infant birth outcomes, such as reducing severe maternal morbidities, low birth weights, and cesarean section births, among others. While much research focuses on the additional medical costs associated with these adverse outcomes, a broader view of benefits from preventive health services would also include monetary estimates of improvements to the quality of life for mothers and children as well as estimates of the value of a statistical life to the extent that infant or maternal deaths are prevented.

Estimates of the costs associated with adverse birth outcomes can be found in several studies. Phibbs et al. (2022) estimated that the additional medical costs in California for severe maternal morbidity events have been reported to be, on average, $7644 once physician and readmission costs are added to the hospital costs. Similar estimates for women in New York City with at least one SMM have been reported by Howland et al. (2018). These costs vary significantly according to the type of severe morbidity—patients with cardiac arrest during delivery have additional medical costs of almost $87,000 (Black et al., 2021). Chen et al. (2018) report that the additional hospital costs are ten times greater when women have five or more SMM. Another recent study of the economic consequences of severe acute maternal outcomes assigns a monetary value to preventing cardiac events and amniotic fluid embolisms (O’Neil et al., 2021).

Another focus has been on the costs associated with the medical care of premature infants. Westley and He (2017) estimated the additional hospital cost associated with preterm births before 37 weeks of pregnancy and reported costs of $24,583 for the infant and an additional $3078 for maternal health care costs. For infants born at weights below 1500 g, these costs ($145,410 and $8592, respectively) were much higher.

Because most low-income pregnant women have their birthing costs covered by the public Medicaid program, reductions in costly adverse outcomes represent savings to taxpayers. The estimates of health care costs associated with policy-alterable adverse events can be combined with estimates of the effectiveness of doulas to calculate economic impact. Two examples include Kozhaminnil et al. (2016) and Greiner et al. (2019). Kozhimannil et al. (2016) obtained cost estimates from insurance data for the impact of doula support on preterm birth and cesarean delivery. Using a break-even analysis, they found that doulas may generate taxpayer savings if they cost between $929 and $1047 in 2012 dollars ($1183–$1333 in 2022 dollars).

Greiner et al. (2019) incorporated reductions in maternal deaths using simulation analyses with estimates from the literature to predict and assign monetary values to reductions in c-sections, maternal deaths, and other health problems related to delivery associated with a large-scale national rollout of doulas to serve a national theoretical cohort of 1.6 million laboring women experiencing their first births. Assuming doulas were available to serve this extensive group, the authors predict that doula care could reduce c-sections by over 200,000 per year, leading to 46 fewer maternal deaths related to c-section complications. They also predict that doula care will reduce severe morbidity issues such as uterine ruptures and hysterectomies. After assigning monetary values to the reductions in deaths and other health complications, their cost–benefit analysis suggests that doula care could generate societal benefits greater than costs if the doulas were to be paid $1360 or less in 2018 dollars (equal to $1583 in 2022 dollars). While the simulation in Greiner et al. (2019) estimates the reductions in maternal deaths due to a decrease in severe morbidity events, to date, no empirical study of doula care has reported a reduction in maternal deaths. Nevertheless, the evidence on reductions in preterm births, c-sections, and severe morbidity events suggests that doula care targeted towards Black women in low-income communities, as well as made more readily available to other Black women at scale, may have the potential to reduce mortality rates.

## Innovative Uses of Cost–Benefit Analysis to Expand Preventive Interventions

The typical approach to cost–benefit analysis (CBA) is to add all societal benefits arising from the expansion of doula care and compare these benefits to doula costs. We focus on two newer applications of CBA that (1) facilitate public/private partnerships at the state or local level to expand funding through social impact financing for preventive interventions that are likely to generate taxpayer savings and (2) target inequities more directly by only considering benefits that accrue to populations placed at risk due to structural racism at a local or regional level rather than society at large. The latter community-based approach is currently in use by a few innovative philanthropies but could also serve as a more general framework for incorporating equity into government cost–benefit analyses. A recent executive order issued in 2021 by the Biden administration (Executive Order 13,985) requests federal agencies to advance fairness and promote equitable delivery of services. Advancing racial equity and support for underserved communities could be adopted as explicit goals of state and local governments.

How can prevention researchers and policymakers use evidence on the effectiveness of doula care to encourage the expansion of funding for doulas? In addition to the use of Medicaid reimbursements allowed by a small number of states, we suggest two additional financing mechanisms that take advantage of evidence of the effectiveness of doula care.

### Pay for Success

Pay for Success (PFS) is a social impact financing model where private investors provide upfront capital to fund effective preventative interventions that are expected to improve well-being while generating cost savings for the public sector. The government involved in a typical PFS transaction then uses its cost savings to repay the investors with interest, contingent on the program performance on pre-defined metrics measured through a rigorous evaluation. A detailed summary of the first 25 PFS contracts in the USA can be found in Nonprofit Finance Fund (2019). As of 2021, the Social Finance Impact Bond Global Database^2^ reports that 138 social impact financing arrangements have been launched during the last decade, with another nearly seventy of them in some stage of development.

The “what works” movement has identified many proven or promising preventive interventions that can improve people’s lives. A subset of these programs are effective and may save governments more money than they cost (Hendren & Sprung-Keyser, 2020). Some recent examples of interventions that may be cost-saving include recidivism prevention, supportive housing, employment programs, and prenatal and early childhood interventions. While, to date, PFS initiatives have not been directed towards the expansion of doula care, relevant examples of how PFS is being used to reduce racial gaps in preterm births and other public health outcomes are described in Lantz et al. (2016) and Nonprofit Finance Fund (2019). Social impact bond initiatives in South Carolina and Michigan have already been launched to fund the trained nurses to conduct home visits for pregnant women or new mothers. Similar initiatives could be launched using doulas. More discussion of how PFS could be utilized for doula care is discussed by Kadam et al. (2018).

Who are the parties in a Pay for Success social impact financing deal? The stakeholders involved in the typical PFS program include one or perhaps two levels or divisions of government, typically local or state governments in the USA. Private investors such as investment banks and philanthropic foundations offer upfront working capital to provide preventive services over several years. An intermediary organization oversees the deal and makes sure that the private funds are used to provide the agreed-upon services. The intermediary oversees the independent evaluator that conducts a rigorous impact evaluation to determine if the outcomes were achieved. Nonprofit service providers deliver the program services to the target population.

To the extent that doula care for Medicaid-eligible women can be cost-effective, a social impact financing method with a PFS contract might be feasible. Nationally, over 40% of births in the USA are funded by Medicaid, and in a few states with larger disadvantaged populations, Medicaid covers over 60% of all births. Given concerns about poor birth outcomes for Black women and infants in the USA, expanding doula care to these populations could potentially generate sizeable cost savings for state taxpayers. For example, a private funder could invest $2 to $10 million to expand community-based doula care. An impartial evaluator would conduct a rigorous evaluation to determine whether the provision of doula care made a difference in health outcomes by comparing rates of low birth weight or severe morbidity events for the doula-treated women vs. a comparison group. One attractive feature of social impact financing through PFS contracts is that the private investors bear the risk of loss, as opposed to the state or local taxpayers, if the scaled-up doula services fail to meet expectations according to pre-determined health outcome metrics. With private investors providing the working capital, PFS is viewed as a risk-free way for the participating government to expand the doula care program. Private investors could contribute additional funding to expand the doula workforce in the states that already allow partial reimbursement from Medicaid. Given significant racial disparities in maternal health outcomes, PFS financing to expand doula care could reduce health inequities in Black communities.

The PFS arrangement, however, involves a significant amount of legal contracting in advance of service delivery (Nonprofit Finance Fund, 2019). Evaluation costs can also be sizeable. Prior to the contracting process, economic evaluation informs the decisions regarding what outcomes are likely to generate public cost savings and what level of desired outcomes will trigger the successful payments to the private investors.

### Data-Driven Philanthropy

While PFS focuses on the importance of taxpayer savings to promote private funding for preventive interventions, innovative organizations such as The Robin Hood Foundation in New York City, The Constellation Fund in Minneapolis, and The Tipping Point in San Francisco are examples of philanthropies that use cost–benefit analyses tools to guide their grant-making decisions. For these data-driven philanthropies, the only benefits that matter in their calculations are the benefits to the low-income community members. The existence of state or federal tax savings is not considered.

Both the Robin Hood Foundation and the Constellation Fund use a metrics framework for assessing the impact of poverty-fighting interventions on the community. The staff review published research studies to help understand the likely impact of the intervention (in this case, the provision of doula care) on the residents of a particular low-income community. To estimate the impact, they try to estimate the counterfactual (i.e., the expected outcome in the absence of the program), if needed, to estimate the true impact of a program (Weinstein & Bradburd, 2013). The estimated impact is then multiplied by the dollar benefit associated with one unit of impact and the number of participants covered by the program in the community to obtain the total benefits from the intervention. When possible, local data are used to estimate the likely benefits. If an intervention is associated with multiple outcomes, e.g., improved health for mothers and children, then the benefits are added to calculate the total benefits of the program.

We show an example of a metric used by Constellation Fund to estimate the benefits of doula care to high-poverty communities in the Twin Cities. Unlike the Pay for Success initiatives involving social impact bonds, here, the existence of cost savings to taxpayers is not of interest. How can doulas improve the lives in a Black community? The Constellation Fund created a metric below that assumes that a primary benefit to community members is the improved well-being of children who avoid being born at low birth weights. The impact of doula care on low birth weight is translated into dollar terms. The equation used in the metric is as follows:

(# women served) × (% women getting assistance solely because of the program) × ( % avoided low birth weight or preterm births due to the intervention) × (# QALY increase) × ($ QALY).

This equation estimates in dollar terms the improvement in the quality of life for children who avoid being born with low birth weight as a result of doula care. Assume one pregnant woman is offered doula care, and this woman would not have been treated by a doula otherwise. The next step in using the metric is to use estimates from the literature on the likely causal impact of providing doula care on avoiding low birth weight. While estimates vary, a starting point might be the estimated 6.5 percentage point reduction associated with doulas from Gruber et al. (2013) study. Since the focus is on improving the well-being of a child who avoids being born low-weight rather than medical cost savings for taxpayers, only the health benefits to children are of interest here. The typical approach is to measure the impact on a health utility, a measure that describes the status of a person’s health and ranges between 0 (for death) and 1 (for perfect health).

Relative to a perfectly healthy child whose health utility is assumed to be 1.00, health utilities range from 0.50 for children born with severe disabilities to 0.99 for some preterm births. One representative estimate from the literature is from Johnson and Schoeni (2011), who estimate a low-birth-weight penalty in terms of quality of life of 0.0377. In other words, a child born with low birth weight has a health utility of approximately 0.96. Assuming this child lives for 75 years, and the value of one Quality-Adjusted Life Years (QALY) is worth $50,000, avoidance of low birth weight is worth $2000 per year in terms of the quality of the child’s life. Added up over 75 years and discounted back to the time of birth using a discount rate of 3%, the improvement in the quality of life in dollar terms is $61,186. Assuming that doula care can reduce the rate of low birth weight by 6.5 percentage points, hiring a doula is worthwhile if the cost is less than 0.065*61,186 or $3977. This estimate is obtained by replacing the equation above with the following specific assumptions for one woman served who would not otherwise have received care: (1) × (100%) × (6.5%) × ($61,186) = $3977. In practice, a larger number of women would be served, and the metric generates a monetary estimate for one way that doulas benefit the community. Awareness of this higher break-even amount could be used to justify significantly higher compensation for doulas.

While the Constellation Fund metric-based approach, so far, only employs this single metric to evaluate the benefits to the community of doula care, additional metrics could be used. Estimates from the literature on the QALY improvement associated with reductions in various severe maternal morbidity events could be estimated following the above approach. Moreover, while no study has directly linked doula care to the prevention of maternal deaths yet, the fact that the value of a statistical life is more than $10 million might make doula care worthwhile even if the effect of doulas on preventing maternal death is very, very small.

Unlike the Pay for Success model, the data-driven philanthropy approach only considers the benefits accrued to the program participants, who are primarily individuals below 185% of the federal poverty guideline. While a complete societal benefit–cost analysis would incorporate both tax savings and improvements in the well-being of community members, for these two innovative uses of CBA, there are clear restrictions on the definition of whose benefits matter or who has standing. In the data-driven philanthropy approach, selecting the community that will be served is a practical application of incorporating equity considerations into using CBA.

In addition to the question of whose benefits matter, another difference between the two approaches is the size of projects funded. PFS funds typically support large-scale projects, often at the state or county level, with the average funding of the first 25 PFS projects in the USA estimated to be around $9.6 million (Nonprofit Finance Fund, 2019). On the contrary, projects funded by the data-driven philanthropy model are often targeted more narrowly to help expand services provided by nonprofits serving disadvantaged neighborhoods, typically in the range of $50,000 to $400,000 per year. A recent discussion of issues involved in measuring structural racism points out that communities affected by racism may not be well represented by state or county boundaries (Hardeman et al., 2022), which suggests that the ability to target preventive services more narrowly to communities is important. Another difference is in the role of impact evaluation. In PFS deals, impartial third-party observers estimate the effect of the provided services on pre-specified outcomes by gathering outcomes data after the program is implemented. For data-driven philanthropy, no formal ex-post impact evaluation occurs. Instead, funding is allocated based on the predicted likelihood of affecting desired outcomes.

## Discussion

Doula care is a preventive intervention that has strong potential to reduce racial disparities in healthy by impacting severe maternal morbidities and mortality among women in Black communities. While some US states already provide funding through Medicaid for doula care for low-income women, we discussed two other funding mechanisms to expand doula care: Pay for Success through social impact financing and data-driven philanthropy.

While using social impact financing through PFS contracts is a promising approach, it has several challenges to its implementation. First, it involves complicated legal contracts to outline the role of various parties involved (private investors, service providers, intermediaries, evaluators, and government payors). Second, while recent US federal legislations make the use of state Medicaid savings easier to tap for use as success payments to repay private investors, setting aside state or local revenues in the budget to repay PFS investors can be a challenge, as it requires explicit legislative action for setting up an escrow account or sinking fund (Kadam et al., 2018).

The fact that the primary outcome of importance in Pay for Success deals is savings to taxpayers prioritizes the funding of some interventions over others in ways that may not be consistent with maximizing the overall benefits to society or the benefits to the communities most affected by structural racism. The spending programs that are to be scaled up through PFS financing may be those that are the best fit for the financial mechanism (Temple et al., 2015) but may not be of the highest priority to the community being served. After a decade of learning from social impact financing via PFS contracts, there is growing interest in approaches where private funders are willing to pay for desirable outcomes that do not involve repayments based on cost savings and often do not involve an impact evaluation utilizing a comparison group (Fox & Morris, 2021). The first PFS contract that involved payments to investors for desirable results not directly associated with cost savings was the $17 million investment by Goldman Sachs to expand a high-quality preschool program in Chicago (Temple & Reynolds, 2015).

What other benefits of doula care might society value in addition to taxpayer savings? Reducing racial disparities may be an important goal for society, but by itself may not be entirely monetizable. Taxpayers benefit from reductions in public health costs, but these benefits are not weighted in evaluations according to the race of the mothers and children who benefit from expanded services. To the extent that maternal and child well-being and mortality can be reduced by the expansion of doula services, especially for Black women, the benefits to society of doula care may significantly exceed the costs even if these benefits are not included in taxpayer savings.

While data-driven philanthropy operates on a smaller and more local scale than social impact financing through PFS contracts, this approach more directly focuses on equity and efficiency in allocating spending to support community needs. The data-driven approach allocates private funds based on metrics predicting the benefit–cost ratios of health, social, and educational services for residents of poor neighborhoods (typically with residents living at or below 185% of the poverty line). While this type of impact financing has attracted much less academic attention, it may be a promising way of targeting funds to alleviate poverty while also directly impacting racial disparities at the community level. A broader community-based approach to expanding the doula workforce could also help non-Medicaid-eligible Black women access doula care because, as noted by Kozhimannil et al. (2015), even insured women with doula coverage may not be aware of these options.

## Conclusion

In this paper, we presented evidence about racial disparities in maternal health outcomes and discussed the recent literature in public health that links these disparities to the causal influence of structural racism rather than race. Importantly, we show how prevention researchers can leverage translational research by demonstrating to policymakers how private and public partnerships can help expand the funding of doula care to marginalized populations. Doula care is used as an important illustrative example, but our discussion of funding mechanisms can apply more broadly to various preventive interventions. The Pay for Success approach has already been used to expand the nurse home-visiting programs with an important objective of reducing racial gaps in preterm births. The approach using data-driven philanthropy to target giving to communities that may benefit most from expansions of public health spending is especially relevant for honing in on community or neighborhood disparities. Through this financing mechanism, philanthropic funding in the Twin Cities has expanded access to doula care.

This paper provides an economic framework to convince policymakers to make sustainable investments in maternal health by expanding access to doula care. The two funding mechanisms discussed rely on economic evaluation and public/private partnerships. Prevention researchers and policymakers can use both in their quest to enact policies that may promote equitable and sustainable change for residents of Black communities and provide guidance on how equity can be more directly incorporated into financing preventative programs.

## Footnotes

1 As this article goes to press, several other states including CA, MI, NJ, and RI are considering or are starting to allow Medicaid reimbursement for doulas. Discussion of recent legislative language involving doulas is discussed in Ogunwole et al. (2022).

2 https://sibdatabase.socialfinance.org.uk/ accessed on December 6, 2021.

## References

[CR1] Alkema, L., Chou, D., Hogan, D., Zhang, S., Moller, A. B., Gemmill, A., Fat, D. M., Boerma, T., Temmerman, M., Mathers, C., & Say, L. (2016). A systematic analysis by the un Maternal Mortality Estimation Inter-Agency Group. *The Lancet,**387*(10017), 462–474. 10.1016/S0140-6736(15)00838-7 10.1016/S0140-6736(15)00838-7 PMC5515236 26584737

[CR2] APHA. (2020). *Structural racism is a public health crisis: Impact on the Black community*. American Public Health Association. Retrieved December 1, 2021, from https://apha.org/Policies-and-Advocacy/Public-Health-Policy-Statements/Policy-Database/2021/01/13/Structural-Racism-is-a-Public-Health-Crisis

[CR3] Black, C. M., Vesco, K. K., Mehta, V., Ohman-Strickland, P., Demissie, K., & Schneider, D. (2021). Costs of severe maternal morbidity in US commercially insured and Medicaid populations: An updated analysis. *Women’s Health Reports,**2*(1), 443–451. 34671765 10.1089/whr.2021.0026 PMC8524749

[CR4] Bohren, M. A., Berger, B. O., Munthe‐Kaas, H., & Tunçalp, Ö. (2019). Perceptions and experiences of labour companionship: a qualitative evidence synthesis. *Cochrane Database of Systematic Reviews*, (3). 10.1002/14651858.CD012449.pub2 PMC6422112 30883666

[CR5] Bridges, K. M. (2020). Racial disparities in maternal mortality. *New York University Law Review,**95*(5), 1229–1318.

[CR6] Campbell, D. A., Lake, M. F., Falk, M., & Backstrand, J. R. (2006). A randomized control trial of continuous support in labor by a lay doula. *Journal of Obstetric, Gynecologic & Neonatal Nursing,**35*(4), 456–464. 10.1111/j.1552-6909.2006.00067.x 16881989

[CR7] Chen, H.-Y., Chauhan, S. P., & Blackwell, S. C. (2018). Severe maternal morbidity and hospital cost among hospitalized deliveries in the United States. *American Journal of Perinatology,**35*(13), 1287–1296. 29723900 10.1055/s-0038-1649481

[CR8] Chinn, J. J., Eisenberg, E., Artis Dickerson, S., King, R. B., Chakhtoura, N., Lim, I. A. L., Grantz, K. L., Lamar, C., & Bianchi, D. W. (2020). Maternal mortality in the United States: Research gaps, opportunities, and priorities. *American Journal of Obstetrics and Gynecology*. 10.1016/j.ajog.2020.07.021 32682858 10.1016/j.ajog.2020.07.021 PMC7564012

[CR9] Crear-Perry, J., Correa-De-Araujo, R., Lewis Johnson, T., Mclemore, M. R., Neilson, E., & Wallace, M. (2021). Social and structural determinants of health inequities in maternal health. *Journal of Women’s Health,**30*(2), 230–235. 10.1089/jwh.2020.8882 33181043 10.1089/jwh.2020.8882 PMC8020519

[CR10] Crowley, M., & Jones, D. (2017). A framework for valuing investments in a nurturing society: Opportunities for prevention research. *Clinical Child and Family Psychology Review,**20*(1), 87–103. 10.1007/s10567-017-0228-3 28247294 10.1007/s10567-017-0228-3 PMC5396060

[CR11] Declercq, E. R., Sakala, C., Corry, M. P., Applebaum, S., & Herrlich, A. (2014). Major survey findings of listening to mothers SM III: Pregnancy and birth . In *The Journal of Perinatal Education*, 23(1). 10.1891/1058-1243.23.1.9 10.1891/1058-1243.23.1.9 PMC3894594 24453463

[CR12] Everson, C. L., Cheyney, M., & Bovbjerg, M. L. (2018). Outcomes of care for 1,892 doula-supported adolescent births in the United States: The DONA International Data Project, 2000 to 2013. *The Journal of Perinatal Education,**27*(3), 135–147. 10.1891/1058-1243.27.3.135 30364259 10.1891/1058-1243.27.3.135 PMC6193361

[CR13] Falconi, A. M., Bromfield, S. G., Tang, T., Malloy, D., Blanco, D., Disciglio, R. S., & Chi, R. W. (2022). Doula care across the maternity care continuum and impact on maternal health: Evaluation of doula programs across three states using propensity score matching. *EClinicalMedicine,**50*, 101531. 35812994 10.1016/j.eclinm.2022.101531 PMC9257331

[CR14] Fox, C., & Morris, S. (2021). Evaluating outcome-based payment programmes: Challenges for evidence-based policy. *Journal of Economic Policy Reform,**24*(1), 61–77. 10.1080/17487870.2019.1575217

[CR15] Gad, M. M., Elgendy, I. Y., Mahmoud, A. N., Saad, A. M., Isogai, T., Mathias, I. S., Rameez, R. M., Chahine, J., Jneid, H., & Kapadia, S. R. (2021). Disparities in cardiovascular disease outcomes among pregnant and post-partum women. *Journal of the American Heart Association,**10*(1), 1–15. 10.1161/JAHA.120.017832 10.1161/JAHA.120.017832 PMC7955477 33322915

[CR16] Gebel, C., & Hodin, S. (2020). Expanding access to doula care: State of the Union. Retrieved December 5, 2022, from https://www.mhtf.org/2020/01/08/expanding-access-to-doula-care/

[CR17] Gjerdingen, D. K., McGovern, P., Pratt, R., Johnson, L., & Crow, S. (2013). Post-partum doula and peer telephone support for post-partum depression: A pilot randomized controlled trial. *Journal of Primary Care & Community Health,**4*(1), 36–43. 10.1177/2150131912451598 23799688

[CR18] Gomez, A. M., Arteaga, S., Arcara, J., Cuentos, A., Armstead, M., Mehra, R., Logan, R. G., Jackson, A. V., & Marshall, C. J. (2021). “My 9 to 5 Job Is Birth Work”: A case study of two compensation approaches for community doula care. *International Journal of Environmental Research and Public Health,**18*(20), 10817. 34682558 10.3390/ijerph182010817 PMC8535486

[CR19] Greenwood, B. N., Hardeman, R. R., Huang, L., & Sojourner, A. (2020). Physician–patient racial concordance and disparities in birthing mortality for newborns. *Proceedings of the National Academy of Sciences, 117*(35), 21194–21200. 10.1073/pnas.1913405117 PMC7474610 32817561

[CR20] Greiner, K. S., Hersh, A. R., Hersh, S. R., Remer, J. M., Gallagher, A. C., Caughey, A. B., & Tilden, E. L. (2019). The cost-effectiveness of professional doula care for a woman’s first two births: A decision analysis model. *Journal of Midwifery and Women’s Health,**64*(4), 410–420. 10.1111/jmwh.12972 31034756 10.1111/jmwh.12972

[CR21] Gruber, K. J., Cupito, S. H., & Dobson, C. F. (2013). Impact of doulas on healthy birth outcomes. *The Journal of Perinatal Education,**22*(1), 49–58. 10.1891/1058-1243.22.1.49 24381478 10.1891/1058-1243.22.1.49 PMC3647727

[CR22] Hans, S. L., Edwards, R. C., & Zhang, Y. (2018). Randomized controlled trial of doula-home-visiting services: Impact on maternal and infant health. *Maternal and Child Health Journal,**22*(1), 105–113. 10.1007/s10995-018-2537-7 29855838 10.1007/s10995-018-2537-7 PMC6153776

[CR23] Hardeman, R. R., Homan, P. A., Chantarat, T., Davis, B. A., & Brown, T. H. (2022). Improving the measurement of structural racism to achieve antiracist health policy. *Health Affairs,**41*(2), 179–186. 35130062 10.1377/hlthaff.2021.01489 PMC9680533

[CR24] Hardeman, R. R., & Karbeah, J. (2020). Examining racism in health services research: A disciplinary self-critique. *Health Services Research,**55*(S2), 777–780. 10.1111/1475-6773.13558 32976632 10.1111/1475-6773.13558 PMC7518806

[CR25] Hendren, N., & Sprung-Keyser, B. (2020). A unified welfare analysis of government policies. In *Quarterly Journal of Economics, 135*(3), 1209–1318. National Bureau of Economic Research. 10.1093/qje/qjaa006

[CR26] Howland, R. E., Angley, M., Won, S. H., Wilcox, W., Searing, H., & Tsao, T.-Y. (2018). Estimating the hospital delivery costs associated with severe maternal morbidity in New York City, 2008–2012. *Obstetrics & Gynecology,**131*(2), 242–252. 29324605 10.1097/AOG.0000000000002432

[CR27] Janevic, T., Zeitlin, J., Egorova, N., Hebert, P. L., Balbierz, A., & Howell, E. A. (2020). Neighborhood racial and economic polarization, hospital of delivery, and severe maternal morbidity. *Health Affairs,**39*(5), 768–776. 10.1377/hlthaff.2019.00735 32364858 10.1377/hlthaff.2019.00735 PMC9808814

[CR28] Johnson, R. C., & Schoeni, R. F. (2011). The influence of early-life events on human capital, health status, and labor market outcomes over the life course. *The BE Journal of Economic Analysis & Policy*, *11*(3). 10.2202/1935-1682.2521 PMC3569741 23412970

[CR29] Kadam, A., Swanson, J., Kretschmann, K., Lee, M., & Varshney, N. (2018). *Pay for Success: A roadmap for implementation in Minnesota*. Retrieved November 20, 2022, from https://conservancy.umn.edu/bitstream/handle/11299/198095/Pay%20for%20Success-Capstone%20Report.pdf?sequence=1&isAllowed=y

[CR30] Kozhimannil, K. B., & Hardeman, R. R. (2016). Coverage for doula services: How state Medicaid programs can address concerns about maternity care costs and quality. *Birth,**43*(2), 97–99. 10.1111/birt.12213 27160375 10.1111/birt.12213 PMC5530734

[CR31] Kozhimannil, K. B., Hardeman, R. R., Alarid-Escudero, F., Vogelsang, C. A., Blauer-Peterson, C., & Howell, E. A. (2016). Modeling the cost-effectiveness of doula care associated with reductions in preterm birth and cesarean delivery. *Birth,**43*(1), 20–27. 10.1111/birt.12218 26762249 10.1111/birt.12218 PMC5544530

[CR32] Kozhimannil, K. B., Almanza, J., Vogelsang, C. A., & Hardeman, R. R. (2015). Medicaid coverage of doula services in Minnesota: Preliminary findings from the first year. *Interim Report to the Minnesota Department of Human Services*. University of Minnesota. December 2015.

[CR33] Lantz, P. M., Low, L. K., Varkey, S., & Watson, R. L. (2005). Doulas as childbirth paraprofessionals: Results from a national survey. *Women’s Health Issues,**15*(3), 109–116. 15894196 10.1016/j.whi.2005.01.002

[CR34] Lantz, P. M., Rosenbaum, S., Ku, L., & Iovan, S. (2016). Pay for success and population health: Early results from eleven projects reveal challenges and promise. *Health Affairs,**35*(11), 2053–2061. 27834246 10.1377/hlthaff.2016.0713

[CR35] Leonard, S. A., Main, E. K., Scott, K. A., Profit, J., & Carmichael, S. L. (2019). Racial and ethnic disparities in severe maternal morbidity prevalence and trends. *Annals of Epidemiology,**33*(4), 30–36. 10.1016/j.annepidem.2019.02.007 30928320 10.1016/j.annepidem.2019.02.007 PMC6502679

[CR36] Liese, K. L., Mogos, M., Abboud, S., Decocker, K., Koch, A. R., & Geller, S. E. (2019). Racial and ethnic disparities in severe maternal morbidity in the United States. *Journal of Racial and Ethnic Health Disparities, 6*(4), 790–798. 10.1007/s40615-019-00577-w 30877505

[CR37] MacDorman, M. F., Declercq, E. R., & Thoma, M. E. (2017). Trends in maternal mortality by sociodemographic characteristics and cause of death in 27 states and the District of Columbia. *Obstetrics and Gynecology,**129*(5), 811–818. 10.1097/AOG.0000000000001968 28383383 10.1097/AOG.0000000000001968 PMC5400697

[CR38] Mathews, T. J., Macdorman, M. F., & Thoma, M. E. (2015). Infant mortality statistics from the 2013 period linked birth/infant death data set. In *National Vital Statistics Reports,* *64*(9), 1–30. Retrieved December 2, 2022, from https://stacks.cdc.gov/view/cdc/32752 26270610

[CR39] McComish, J. F., Groh, C. J., & Moldenhauer, J. A. (2013). Development of a doula intervention for post-partum depressive symptoms: Participants’ recommendations. *Journal of Child and Adolescent Psychiatric Nursing: Official Publication of the Association of Child and Adolescent Psychiatric Nurses, Inc*, *26*(1), 3–15. 10.1111/jcap.12019 23351103

[CR40] Miller, E. C., Zambrano Espinoza, M. D., Huang, Y., Friedman, A. M., Boehme, A. K., Bello, N. A., Cleary, K. L., Wright, J. D., & D’Alton, M. E. (2020). Maternal race/ethnicity, hypertension, and risk for stroke during delivery admission. *Journal of the American Heart Association*, *9*(3), e014775. 10.1161/JAHA.119.014775 10.1161/JAHA.119.014775 PMC7033883 31973601

[CR41] Minnesota Department of Health. (2015). Infant mortality reduction plan for Minnesota, part one. Saint Paul, MN. Retrieved December 1, 2021, from https://www.health.state.mn.us/docs/people/womeninfants/infantmort/infantmortality.pdf

[CR42] National Academies of Sciences and Medicine, E. (2016). *Advancing the power of economic evidence to inform investments in children, youth, and families*. Washington DC: The National Academies Press. 10.17226/23481 27536749

[CR43] Nonprofit Finance Fund. (2019). *Pay for Success: The First 25*. Retrieved November 20, 2021, from https://nff.org/report/pay-success-first-25

[CR44] Ogunwole, S. M., Bozzi, D. G., Bower, K. M., Cooper, L. A., Hardeman, R., & Kozhimannil, K. (2022). Health equity considerations in state bills related to doula care (2015–2020). *Women's Health Issues*. 10.1016/j.whi.2022.04.004 PMC10224765 35610121

[CR45] O’Neil, S., Platt, I., Vohra, D., Pendl-Robinson, E., Dehus, E., Zephyrin, L., & Zivin, K. (2021). *The high costs of maternal morbidity show why we need greater investment in maternal health*. Mathematica Policy Research.

[CR46] Oregon Health Authority (2022) Public notice, June 8, 2022. Retrieved August 8, 2022, from https://www.oregon.gov/oha/HSD/OHP/Announcements/Doula-Rates0622.pdf

[CR47] Petersen, E. E., Davis, N. L., Goodman, D., Cox, S., Syverson, C., Seed, K., Shapiro-Mendoza, C. K., Callaghan, W. M., & Barfield, W. (2019). Racial/ethnic disparities in pregnancy-related deaths—United States, 2007–2016. *Morbidity and Mortality Weekly Report,**68*(35), 762. 31487273 10.15585/mmwr.mm6835a3 PMC6730892

[CR48] Phibbs, C. M., Kozhimannil, K. B., Leonard, S. A., Lorch, S. A., Main, E. K., Schmitt, S. K., & Phibbs, C. S. (2022). The effect of severe maternal morbidity on infant costs and lengths of stay. *Journal of Perinatology*, 1–6. 10.1038/s41372-022-01343-3 PMC9098672 35184145

[CR49] Platt, T., & Kaye, N. (2020). *Four state strategies to employ doulas to improve maternal health and birth outcomes in Medicaid*. Washington, DC: The National Academy for State Health Policy.

[CR50] Scholars, B. W. (2020). Black maternal health research re-envisioned: Best practices for the conduct of research with, for, and by Black mamas. *Harvard Law & Policy Review,**14*(2), 393–415.

[CR51] Scott, K. A., & Davis, D. A. (2021). Obstetric racism: Naming and identifying a way out of Black women’s adverse medical experiences. *American Anthropologist,**123*(3), 681–684. 10.1111/aman.13559 10.1111/aman.13559

[CR52] Society for Prevention Research. (2020). SPR and NPSC Statement Condemning Racism. Retrieved November 30, 2021, from https://www.preventionresearch.org/spr-and-npsc-statement-condemning-racism/

[CR53] State of New Jersey. (2021). Newsletter: Medicaid/NJ FamilyCare coverage of doula services revised billing codes. *Department of Human Services Division of Medical Assistant & Health Services*, *31*(4). Retrieved December 1, 2022, from https://www.state.nj.us/humanservices/dmahs/info/Newsletter_31-04_Doula.pdf

[CR54] Tangel, V., White, R. S., Nachamie, A. S., & Pick, J. S. (2019). Racial and ethnic disparities in maternal outcomes and the disadvantage of peripartum Black women: A multistate analysis, 2007–2014. *American Journal of Perinatology,**36*(8), 835–848. 10.1055/s-0038-1675207 30396228 10.1055/s-0038-1675207

[CR55] Temple, J. A., Punay, M., Barr, K., & Wagstrom, A. (2015). *Pay-for-performance financing to expand cost-effective social services: Lessons learned in Minnesota*. Nonprofits Assistance Fund, Minneapolis MN. Retrieved December 1, 2022, from https://www.propelnonprofits.org/wp-content/uploads/2017/09/mnpayforperformancefinancing_lessons_report.pdf

[CR56] Temple, J. A., & Reynolds, A. J. (2015). Using benefit-cost analysis to scale up early childhood programs through Pay-for-Success financing. *Journal of Benefit-Cost Analysis,**6*(3), 628–653. 10.1017/bca.2015.54 27882288 10.1017/bca.2015.54 PMC5116808

[CR57] Thomas, S. B., & Casper, E. (2019). The burdens of race and history on black people’s health 400 years after Jamestown. In *American Journal of Public Health,* *109*(10), 1346–1347). American Public Health Association. 10.2105/AJPH.2019.305290 10.2105/AJPH.2019.305290 PMC6727285 31483719

[CR58] Trotter, C., Wolman, W.-L., Hofmeyr, J., Nikodem, C., & Turton, R. (1992). The effect of social support during labour on post-partum depression. *South African Journal of Psychology,**22*(3), 134–139. 10.1177/008124639202200304

[CR59] Van Eijk, M. S., Guenther, G. A., Jopson, A. D., Skillman, S. M., & Frogner, B. K. (2022a). Health workforce challenges impact the development of robust doula services for underserved and marginalized populations in the US. *The Journal of Perinatal Education,**31*(3), 133–141. 36643390 10.1891/JPE-2021-0013 PMC9829116

[CR60] Van Eijk, M. S., Guenther, G. A., Kett, P. M., Jopson, A. D., Frogner, B. K., & Skillman, S. M. (2022b). Addressing systemic racism in birth doula services to reduce health inequities in the United States. *Health Equity,**6*(1), 98–105. 35261936 10.1089/heq.2021.0033 PMC8896213

[CR61] Weinstein, M. M., & Bradburd, R. M. (2013). *The Robin Hood rules for smart giving*. In Columbia University Press.

[CR62] Weiss, R. E. (2021, September 13). *The Cost of Hiring a Doula for Your Pregnancy*. Retrieved December 1, 2021, from https://www.verywellfamily.com/how-much-does-a-doula-cost-4123633

[CR63] Westley, E., & He, L. (2017). *Estimating effects of birth indicators on health care utilization costs and infant mortality: Technical appendix*. Retrieved December 1, 2021, from www.wsipp.wa.gov

[CR64] Wolman, W.-L., Chalmers, B., Hofmeyr, G. J., & Nikodem, V. C. (1993). Post-partum depression and companionship in the clinical birth environment: A randomized, controlled study. *American Journal of Obstetrics and Gynecology,**168*(5), 1388–1393. 8498417 10.1016/S0002-9378(11)90770-4

[CR65] Zhang, J., Bernasko, J. W., Leybovich, E., Fahs, M., & Hatch, M. C. (1996). Continuous labor support from labor attendant for primiparous women: A meta-analysis. *Obstetrics & Gynecology,**88*(4), 739–744. 8841285 10.1016/0029-7844(96)00232-3

[CR66] Zuckerwise, L. C., & Lipkind, H. S. (2017). Maternal early warning systems—Towards reducing preventable maternal mortality and severe maternal morbidity through improved clinical surveillance and responsiveness. *Seminars in Perinatology,**41*(3), 161–165. 28416176 10.1053/j.semperi.2017.03.005

